# Effect of lipid extraction and room temperature transportation of bovine oocytes determined by MRM profiling

**DOI:** 10.21203/rs.3.rs-3788683/v1

**Published:** 2023-12-23

**Authors:** Camila Bruna de Lima, Marcella Pecora Milazzotto, Alessandra Aparecida Vireque, Daniel Carlino Joaquim, Tiago Jose Paschoal Sobreira, Christina Ramires Ferreira

**Affiliations:** Université Laval, Department of Animal Sciences; Universidade Federal do ABC, CCNH; Invitra Assisted Reproductive Technologies Ltd; Invitra Assisted Reproductive Technologies Ltd; Purdue University, Center for Analytical Instrumentation Development; Purdue University, Center for Analytical Instrumentation Development

**Keywords:** MRM profiling, Lipid extraction, Oocytes, Bovine

## Abstract

Lipids play many important physiological roles in mammalian reproduction, being essential for the acquisition of oocyte competence and post-fertilization embryonic development. Lipid profiling in samples of minute size, such as oocytes, is challenging but has been achieved by mass spectrometry technologies such as multiple reaction monitoring (MRM) profiling. With the goals of further simplifying sample workflow and investigating the influence of pre-analytical conditions, we have evaluated how different extraction methods and transportation of lipid extracts in vacuum and at room temperature impacted the lipid profile of bovine oocytes. Using a comprehensive method, 316 MRMs associated with lipids of 10 different classes were screened in oocyte lipid extracts prepared by 2 extraction methods (one-step methanol addition or Bligh and Dyer) and transporting them in dry ice or at room temperature inside vacuum packages. No changes in the multivariate analysis (PCA) were noticeable due to transportation temperature, while lipid profiles were more affected by the lipid extraction protocol. Sample extraction using pure methanol favored the detection of phospholipids uniformly, while Bligh and Dyer favored the detection of neutral intracellular lipids. Triacylglycerol lipids and free fatty acids yielded decreased abundances when samples were transported at room temperature. We conclude that if samples are submitted to the same lipid extraction protocol and same transportation batch at room temperature coupled with vacuum conditions it is possible to analyze lipid extracts of bovine oocytes and still obtain informative lipid profiling results.

## 1 INTRODUCTION

Lipids exhibit a huge structural variety, which explains why they are widely involved in many biological functions. Phospholipids are the building blocks for the formation of cellular membranes in the form of lipid bilayer. Fatty acids also function as structural constituents of membranes, precursors in steroidogenic and eicosanoids pathways. Intracellularly, fatty acids can also be stored within lipid droplets as triacylglycerides (TAG), providing a potent source of energy upon demand. Other subclasses like phosphatidylinositol (PI) and phosphatidylserine can act as secondary messengers in cell signaling processes. Considering these many physiological functions, it is not surprising that the study of lipids is of great importance in reproductive research. Lipid metabolism plays an important role in the gradual acquisition of oocytes developmental competence and ability to be fertilized [1, 2]. Besides, modulation of lipid amounts in the cytoplasm of gametes and embryos has an important impact in the improvement of cryopreservation technologies.

The most common way to ensure efficient extraction of cellular lipids from a wide range of biological samples is to employ a mixture of organic solvents [3]. The extraction methods described by Folch (1957) [4] and Bligh and Dyer (1959) [5] are standard procedures for the recovery of total lipids. Both methods rely on chloroform and methanol to form a monophasic system to extract and dissolve the lipids followed by a purification step through the addition of water, creating a biphasic system that separates polar and non-polar compounds [6]. Although these protocols are well established, sample dilution and pipetting can cause sample loss in microextraction. An alternative single-step lipid extraction procedure uses ethanol or methanol. Cold methanol, for example, is often used as a single extraction solvent due to its intermediate polarity [7] and its use has been reported as a feasible approach for shotgun lipidomics [8, 9].

Metabolic and lipid profiling benefits from the development of increasingly sensitive and selective analytical techniques, mainly those based on mass spectrometry and nuclear magnetic resonance analysis [10, 11]. For lipid analysis from mammalian oocytes and embryos, the application of LC-MS based methods require the pooling of hundreds of samples, which makes this approach unpractical for such minute samples. Therefore, lipidomics studies in reproduction have relied on the direct assessment of the lipid profile in single organisms by desorption electrospray ionization (DESI) and matrix-assisted laser desorption/ionization (MALDI) mass spectrometry [10] and more recently by other technologies that allow trace analysis such as single-cell capillary electrophoresis high-resolution mass spectrometry (CE-HRMS) [12] and MRM-profiling [13].

Given the diversity of chemical properties for lipids, different experimental approaches can be used to isolate and study specific lipids [14]; wherein a considerable amount of errors can be introduced during the preanalytical steps that have detrimental effects on sample quality. In a previous study, Ellervik and Vaught identified sample collection, processing, transport, and storage as the most relevant technical elements impacting the outcomes in metabolomics studies [15, 16]. Still, longitudinal lipidomics and metabolomics studies are being carried out and are able to obtain consistent and informative results.

In general, lipid samples are stored long-term at −80°C and, when necessary, transported in dry ice or under nitrogen gas to prevent oxidative damage. However, in our practice, we observed in a pilot study that good quality profiles could be obtained from lipid extracts transported at room temperature for a short period of time (data not shown). Therefore, we decided to carry out a well-designed study to investigate the effects of storage and transportation in lipid profiling since the possibility of using room temperature would be very cost-effective and advantageous to foster international collaborations and accessibility to different facilities and equipment. Furthermore, we believe that such a study could reassure the potential of lipidomics studies to provide robust results even when preanalytical conditions are not considered ideal.

Finally, using bovine oocytes, which are minute lipid-containing sample, and the MRM-profiling approach as a comprehensive analytical method, we evaluated the impact of two variables on the lipid profiles. The first was the comparison of Bligh and Dyer versus a faster single-step sample processing method using methanol. The second variable analyzed was the impact of 3-day room temperature transportation under vacuum.

## 2 MATERIAL AND METHODS

All culture media and reagents were purchased from Sigma Chemical Company (St. Louis, MO, USA), except where otherwise indicated. Phosphate buffer saline (PBS) solution was supplied by Gibco/BRL (Grand Island, NY, USA). Methanol (ACS/HPLC) grade was purchased from Burdik & Jackson (Muskegon, MI, USA). Ultrapure water, purified by Direct-Q water system (Millipore, Bedford, MA, USA), was used for the preparation of solvents.

### 2.1 Experimental Design

**Figure 1:**
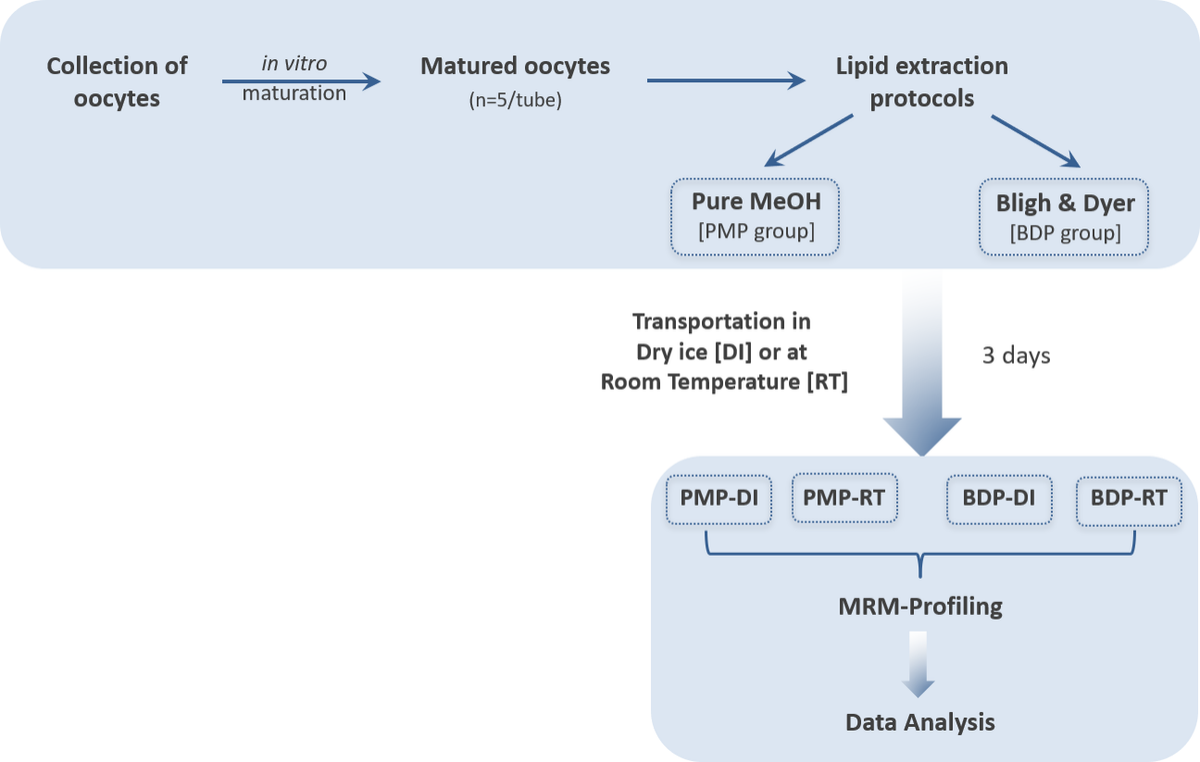
Experimental design used to compare the impact of two lipid extraction methods and room temperature transportation under vacuum vs. dry ice on lipid profiles of *in vitro* matured bovine oocytes.

Collection of bovine MII-stage oocytes and lipid extraction were carried out at Invitra – Assisted Reproductive Technologies Ltd. (Ribeirão Preto, SP, Brazil). Half of the samples were processed using Bligh & Dyer protocol (BDP) exactly as described in a recent publication [13] and the other half was processed by a single-step pure methanol addition (PMP). The lipid extracts were dried under N_2_ stream and stored at −80°C until transportation. Both groups of samples were shipped either on dry ice (DI) or at room temperature (RT) from Brazil to the U.S. (Bindley Biosciences facility at Purdue University (West Lafayette, IN, USA). Thus, the four study groups are: (i) Pure Methanol Protocol – Room Temperature (PMP-RT), (ii) Pure Methanol Protocol – Dry Ice (PMP-DI), (iii) Bligh & Dyer Protocol – Room Temperature (BDP-RT) and (iv) Bligh & Dyer Protocol – Dry Ice (BDP-DI). The total transit time of the samples (i.e. the time during which samples were kept at room temperature or dry ice for transportation) was three days. Upon arrival at the laboratory (USA), all samples were immediately checked out for vacuum maintenance. The room temperature experimental groups were also maintained at −80°C after the transportation. The experimental design is summarized in Fig. 1.

### 2.2 Collection of oocytes

Bovine ovaries were collected from a slaughterhouse and transported to the laboratory in 0.9% physiological saline supplemented with 0.05 g. L^−1^ streptomycin at 35 °C. Antral follicles (3- to 8-mm) from 40 – 50 ovaries/experiment were aspirated with 18 G needles adapted to 20 mL syringes. Cumulus-oocyte complexes (COC) were morphologically evaluated under a stereomicroscope, as previously described [13] and those with at least three compact layers of cumulus cells were selected and washed several times in TCM/HEPES medium supplemented with 50 mg. L^−1^ gentamycin and 0.1% BSA.

### 2.3 In vitro maturation protocol

Pools of 20 *cumulus*-oocyte complex (COCs) were cultured in drops of 100 μL in Petri dishes covered with 3 mL of M8410 mineral oil at 38.8°C for 22 h in a humidified atmosphere containing 5% CO_2_. The maturation medium was HEPES-buffered tissue culture medium-199 (TCM-199, Gibco/BRL, Grand Island, NY, USA) supplemented with 0.2 mM sodium pyruvate, 100 IU.mL^−1^ penicillin G, 100 μg.mL^−1^ streptomycin, 0.5 μg.mL^−1^ follicle stimulating hormone (FSH, Folltropin-Bioniche, Canada), 5 μg.mL^−1^ luteinizing hormone (LH, Lutropin-Bioniche, Canada) and 1 μg.mL^−1^ 17β-estradiol. Fetal bovine serum (FBS, Invitrogen Gibco/BRL) was added to the standard TCM medium at 10% (v/v).

After *in vitro* maturation, oocytes were stripped of *cumulus* cells by gentle successive pipetting in 100 μL drops of 0.5% hyaluronidase over a period of 5 min. Completely denuded and morphologically undamaged oocytes were removed from the microdrops and washed from residual CC three times in a Petri dish containing 200 μL TCM medium supplemented with 5% FBS. Immediately, oocytes were checked for 1st polar body (PB) extrusion to verify that maturation occurred (oocytes in metaphase II stage). Procedures were performed at 38.5°C and immediately after PB analyses oocytes were stabilized during 60 min in IVM medium before sample preparation. Mature oocytes (MII stage) were washed in PBS solution and randomly transferred to 1.5 mL tubes (Axygen, DNase and RNase-free grade) in a minimal volume of 1–2 μL, according to following experimental groups: Pure methanol protocol (PMP), Bligh & Dyer protocol (BDP).

### 2.4 Single-step extraction protocol using pure methanol

Pools of 5 oocytes were washed 3 times in methanol: H_2_O solution (1:3 v/v), transferred to a microtube and immediately covered with 10 μL of pure methanol (*n* = 10 replicates; 5 oocytes/replicate). After the complete evaporation of the solvent (120 min under laminar flow), the microtubes containing the samples assigned to room temperature (RT) or dry ice (DI) groups were vacuum-sealed in a plastic bag and stored in a freezer at −80°C until samples were transported.

### 2.5 Bligh & Dyer extraction protocol

The Bligh & Dyer protocol used for sample extraction was the same as described in our previous publication [11]. In detail, 40 μL of ultrapure water was added to the microtube containing the oocytes (*n* = 10 replicates; 5 oocytes/replicate) and the mixture was gently mixed to promote cell lysis. Then, 50 μL of chloroform and 90 μL of methanol were added and mixed by pipetting for 15 seconds (1st-phase solution). Next, another 50 μL of CHCl_3_ and 50 μL of ultrapure H_2_O were added and the samples were incubated for 5min at room temperature. Samples were then centrifuged at 800 × *g* for 60 sec to enhance polar/nonpolar phase separation (2nd-phase solution). Finally, 80 μL of the nonpolar phase (CHCl_3_) and 80 μL of the polar phase (MeOH/H_2_O) were combined in a new microtube. It is important to notice that only proteins were left behind since the polar and the organic phases were combined to try recovering the most lipid amount of the samples. Combined organic and water phases were dried using N_2_ and the tubes containing the samples, assigned to room temperature (RT) or dry ice (DI) groups, were vacuum-sealed in double packages to ensure sample performance and stored at −80 °C until shipment.

### 2.6 MRM Profiling

MRM profiling is a two-step strategy (discovery step/screening step) for small molecule biomarker identification first reported in 2016 [Ferreira et al., 2016]. For this study, MRM-profiling was performed using the screening methods described by de Lima et al., 2018.

Briefly, the first step consisted of creating a list of MRMs using the combination of the molecular ions mass-to-charge ratios (*m/z*) with the expected diagnostic product ions for each lipid class or fatty acyl residue. Triacylglycerols (TAGs) were monitored by the parent ion of their ammonium adducts and the product ion corresponding to the neutral loss of specific fatty acyl residues [palmitic (C16:0), palmitoleic (C16:1), stearic (C18:0), oleic (C18:1), linoleic (C18:2), arachidic (C20:0) and arachidonic (C20:4)]. Free fatty acids were monitored at the negative ion mode only by the parent ion. The final list with 1,586 entries (corresponding to (14,184 Lipid Maps entries since constitutional isomers were combined into a single entry) was capable of monitoring 10 different classes (PC, SM, PE, PI, PG, PS, TAGs, cholesteryl esters, acyl-carnitines and FFA). The discovery step of MRM-profiling was then performed in representative pools (*n* = 25 oocytes/pool) from each study group.

**Figure 2:**
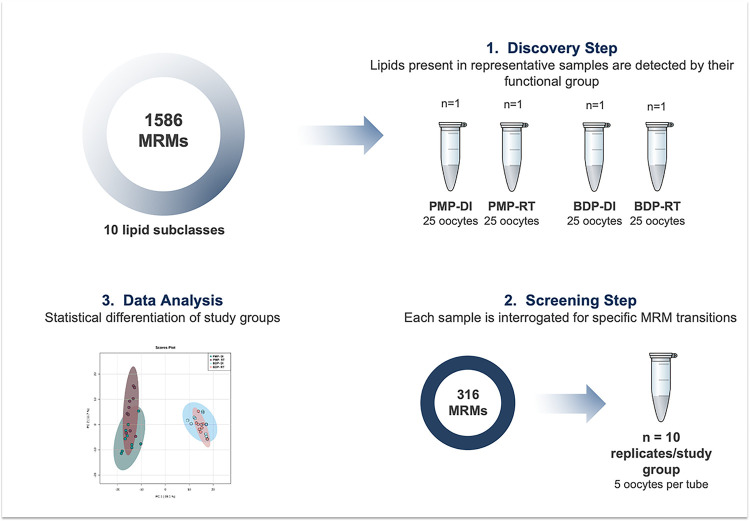
First, 1,586 MRMs from 10 distinct subclasses (PC, SM, PE, PI, PG, PS, TAGs, cholesteryl esters, acyl-carnitines and FFA) were used in the discovery step to interrogate a pool of samples representative of each experimental group. Transitions monitored in this first step were considered representative when absolute ion intensity was at least 50 ions counts over the blank extraction sample. In the second step a total of 316 representative MRMs were selected for profiling (**Supplementary Table 1**). Sets of oocytes from all experimental groups were interrogated in 10 replicates under the same operational conditions.

In the second step, MRM profiling of individual samples was performed (*n* = 10 replicates/group), and the entire sample set was interrogated but this time only for the 316 ion pairs that were detected as higher than in a blank extraction sample during the discovery step. A schematic of the MRM profiling workflow is shown at Fig. 2.

In both steps dry lipid extracts were re-suspended in 20μL of ACN + MeOH + 300mM NH_4_Ac 3:6.65:0.35 (v/v) and a micro-autosampler (G1377A) was used to deliver 8 μL per sample by flow injection to the ionization source of an Agilent 6410 QQQ mass spectrometer (Agilent Technologies, Santa Clara, CA, USA). Two samples injections were necessary to monitor the 316 selected MRMs. The capillary pump of the autosampler operated with a pressure of 150 bar with and a 20 μL/min flow. Capillary voltage was 3.5–5 kV and the gas flow was 5.1 L/min at 300°C.

### 2.7 Statistical Analysis

The ion intensity of each MRM transition was recovered and normalized by the total ion current (TIC). Data analysis using relative ion abundances was performed using the online software Metaboanalyst 4.0 (www.metaboanalyst.ca) for multivariate statistics by principal component analysis (PCA) and also using GraphPad Prism 7 for univariate analysis. A value α = 5% was considered significant for all the comparisons.

## 3 RESULTS

**Figure 3:**
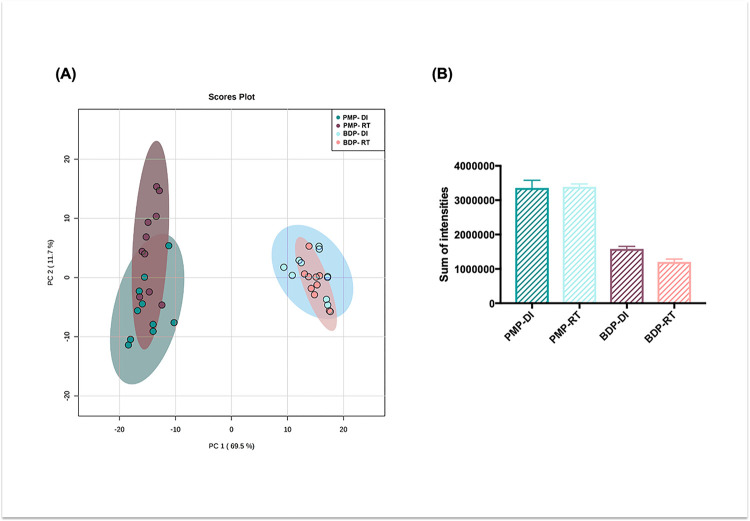
(a) PCA scores plot showing the samples processed according to two extraction methods (PMP - pure methanol protocol, and BDP - Bligh & Dyer protocol) and transported at different temperatures (DI – dry ice and RT – room temperature). PC1 + PC2 was responsible for 81.2% of the variance among the groups (b) Sum of total ion intensities of all studied lipids by experimental group.

When the four experimental groups were analyzed together, two clearly distinct clusters were observed in the PCA scores plot according to the type of lipid extraction protocol used (Fig. 3a). Note that separation due to the temperature of transport (DI vs. RT) of the samples was not observed. Group discrimination due to the temperature of transport (DI *vs.* RT) was also not observed when only PMP or only BDP samples were analyzed by PCA (**Supplementary Figs. 1 and 2**). Besides PCA, we compared the sum of the relative intensities of lipids from all lipid classes according to the treatments and the graph shows the overall signal was higher for pure methanol extraction, compared to the Bligh & Dyer method (Fig. 3b).

### 3.1 Impact of the extraction method on the total ion intensity for each lipid class

**Figure 4:**
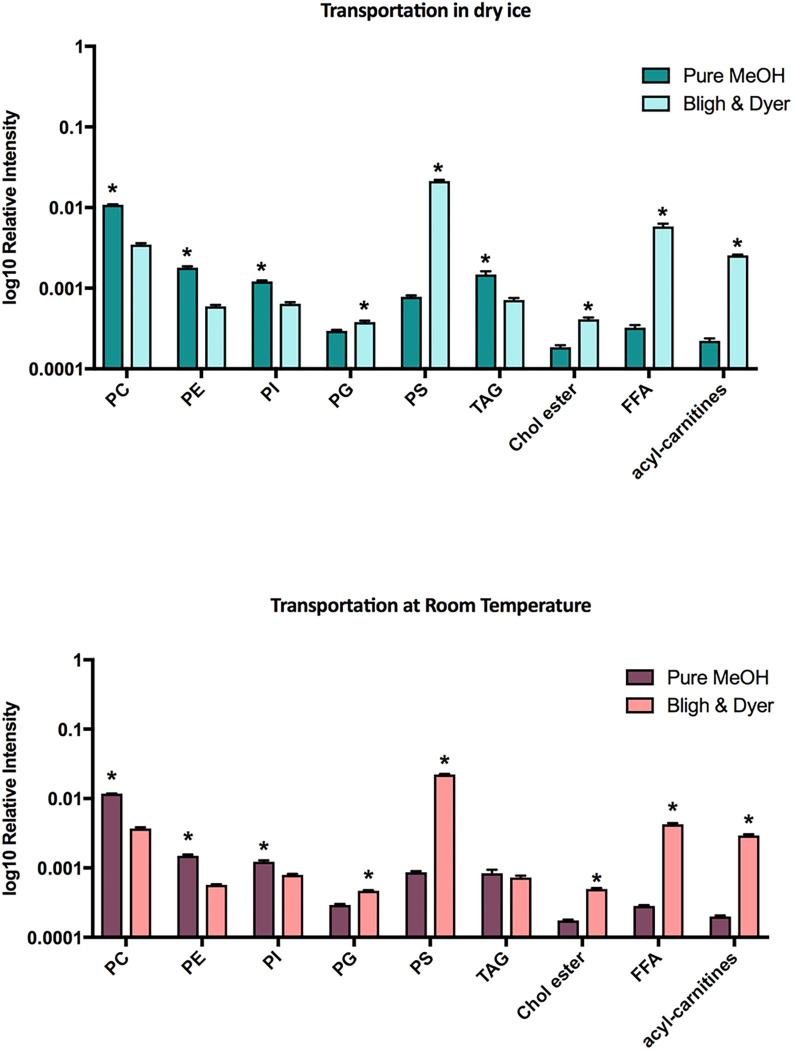
Comparison of the sum of the relative ion intensities between lipid extraction protocols considering transportation in dry ice (upper graph) and at room temperature (bottom graph). Independent of the temperature of transportation, several differences between the processing methods can be observed. * Differences were considered significant when *P*<0.05.

In the scores plot shown in Fig. 3, we observe that the type of lipid extraction method has a more pronounced impact on the lipid profile than the temperature of transportation. When the total ion intensities were plotted for each lipid class (Fig. 4), we observed that the PMP method favored the detection of Phosphatidylcholines (PC) and Sphingomyelins (SM), Phosphatidylethanolamines (PE), Phosphatidylinositol (PI) and TAG, while the Bligh & Dyer protocol facilitated the detection of Phosphatidylglycerol (PG), Phosphatidylserines (PS), Cholesterol esters, Free Fatty Acids (FFA) and acyl-carnitines, regardless of the temperature of sample transportation. Of note, PMP poorly detected PS, FFA and acyl-carnitines.

### 3.2 Impact of sample transportation temperature on the total ion intensities of each lipid class

**Figure 5:**
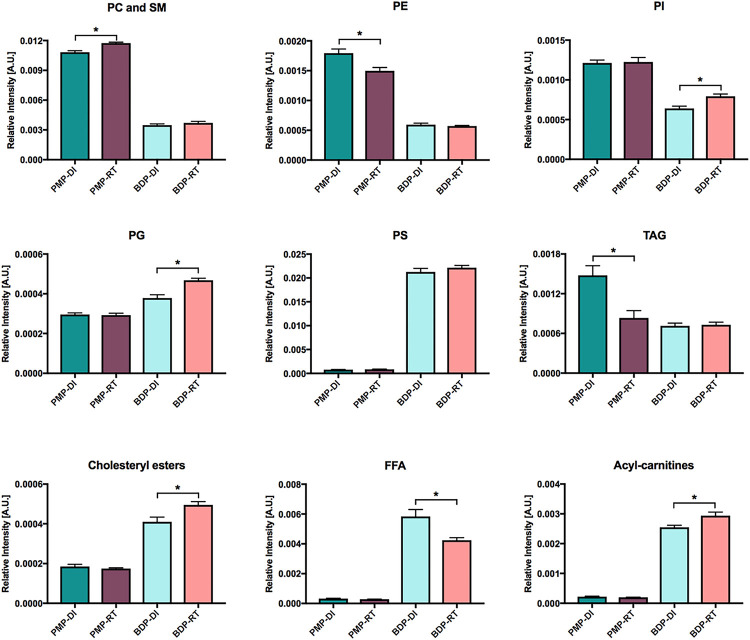
Comparisons between groups transported in dry ice versus room temperature for all subclasses. Pure Methanol Protocol – Dry Ice (PMP-DI), Pure Methanol Protocol – Room Temperature (PMP-RT), Bligh & Dyer Protocol – Dry Ice (BDP-DI) and Bligh & Dyer Protocol – Room Temperature (BDP-RT). * Differences were considered significant when *P*<0.05.

As mentioned previously, the overall lipid profile of samples transported at room temperature inside vacuum packages remained similar to the lipid profile of samples transported in dry ice when data was visualized by PCA. Nonetheless, when we compared only the impact of the transportation method in each lipid subclass, we were able to detect some slight differences. For samples processed by PMP, we observed increased relative amounts of PC/SM at room temperature, but a decrease in PE, and TAGs (Fig. 5). For samples processed by BDP, a higher sum of the relative ion intensities was observed for PI, PG, cholesteryl esters and acyl-carnitines when the samples were transported at room temperature.

Finally, the detection of FFA was inefficient when pure methanol extraction was used. Results for this subclass were substantially improved by the use of Bligh & Dyer method although relative amounts also slightly decreased when samples were transported at room temperature (Fig. 5).

## 4 DISCUSSION

Two lipid extraction protocols, including Bligh & Dyer method and a one-step methanol method, and two temperatures for sample transportation (room temperature or dry ice), were compared to in order to improve pre-analytical procedures of lipid analysis in reproductive cells. Lipid profiles obtained from room temperature or dry ice samples were similar, but they were affected by the extraction methods, when methanol and Bligh & Dyer were compared within RT or DI groups. Yet, it should be noted that the lipid profiles of BDP-RT samples were very comparable to BDP-DI, the classical or reference protocol requiring cold chain maintenance (Bligh & Dyer, 1959).

There is little information available regarding specific protocols that minimize analytical error and variability in samples to be evaluated for their lipid profiles, which is particularly important for minute and valuable samples. In the specific case of reproduction research, samples such as oocytes and embryos may often be from vulnerable populations or represent unique opportunities that would be difficult to replicate (e.g., in assisted reproductive technology procedures and clinical studies on reproductive medicine). Considering the importance of lipid research in reproduction, in the present study, we performed a simplified workflow for lipid analysis and investigated if the extraction method and/or the room temperature transportation could qualitatively impact the lipid recovery and profile, and ultimately the output data obtained from these types of samples.

In general, working with lipids demands careful handling and storage due to their high oxidation and hydrolysis rates [17]. In living organisms, oxidation is a normal process controlled by antioxidants; however, after death, this protective function decreases significantly and results in lipid degradation [18]. Taken into account that lipid oxidation proceeds very slowly at initial stages [19], and even though the time between lipid extraction and data acquisition was short in our study, considerably reducing the chances of oxidation, the samples were also kept in vacuum packaging as a strategy to avoid exposure to oxygen at room temperature.

It is known that the effectiveness of the lipid extraction protocol, to a large extent, depends on the chemical nature of the lipids and the type of associations in which they are found in the cell [20]. Hydrophobic lipids, for example, may be extracted with non-polar solvents such as ethyl ether or chloroform. Membrane associated lipids, on the other hand, require polar solvents such as ethanol or methanol to disrupt the hydrogen bond networks or electrostatic forces between lipids and proteins (Bou Khalil et al., 2010; Saini et al., 2021). Note that for the present study, after Bligh & Dyer extraction, we combined the aqueous and organic phases to recover most of the lipids in the sample.

For bovine oocytes, the choice of the sample processing protocol (one-step methanol *vs.* Bligh & Dyer) impacted the lipid profiles as indicated by PCA. Membrane lipids, including PC, PE and PI lipids, showed higher relative intensities when lipid extraction was done using only with pure methanol, even though the same was not observed for PG and PS lipids. In this way, one-step lipid extraction with methanol for analysis of membrane lipids in oocytes and embryos has been reported successfully and allowed accurate identification of lipid changes within individual lipid classes [22, 23]. Neutral lipids such as acyl-carnitines, cholesteryl esters and FFA were more efficiently extracted when a chloroform: methanol mixture was used. In a complex matrix such as the blood plasma, lipids of all classes can be recovered via chloroform/methanol extraction, but low recovery occurs for charged and non-polar lipids [24]. Another concern regarding lipid analysis is the fact that the samples often need to be stored or transported prior to analysis. Following a “gold standard” practice, lipids should be extracted immediately upon sampling and kept frozen at temperatures ranging from − 20 to −80°C until analysis [25, 26]. In the present study, we evaluated the effect of deviations from the gold standard storage/handling practice by transporting lipid extracts between Brazil and the USA at room temperature. It is important to mention that we did not monitor temperature variations during room temperature transportation, which can be considered as a limitation of this study, but also reinforces the robustness of the technical approach. Even if big temperature variations occurred, they did not have a pronounced impact the lipid profile, as evidenced by the unsupervised PCA analysis.

Although the overall the lipid profile was not impacted by the transportation at room temperature under vacuum, some significant changes were observed between DI and RT when considering the sum of the relative ion intensities for each lipid class. For PMP-RT, significant decreased abundance was observed for occurred for PE and TAGs and an increased abundance was observed for PC/SM compared to PMP-DI. For the BDP-RT protocol, higher relative abundance was observed for PI, PG, cholesteryl esters, and acyl-carnitines and lower abundance for FFA. We speculate that some of these differences are not necessarily related to the transportation at RT which may cause some lipid oxidation, but they might be related to the freeze-thaw cycles to which the samples were submitted before data acquisition. Unlike samples transported on dry ice that were only thawed prior to MS analysis, samples transported at room temperature were exposed to two freeze-thaw cycles. The first one occurred when the lipid extracts were removed from the − 80°C freezer to be shipped at room temperature. Then lipid extracts were stored at −80°C upon arrival. The second freeze-thaw cycle occurred when the lipid extracts were removed from the freezer for MS analysis. More studies are necessary to confirm this hypothesis.

The findings of this study suggest that the extraction method has more influence on the lipid profiling than the sample transportation at room temperature. In this way, considering that the Bligh & Dyer “gold standard” protocol requires maintenance of the cold chain of the samples, the room temperature transport allowed a more effective detection of PI, PG, cholesterol esters and acyl-carnitines, and other evaluated lipid classes, except FFA, in comparison with dry ice. Remarkably, TAG and FFA were the lipid classes most affect by sample transportation temperature, showing lower recovery in oocytes transported at room temperature after methanol or Bligh & Dyer extraction method, respectively.

If all samples in a given experiment are processed in the same way it is possible to obtain informative lipid profiles and consistent results in lipidomics of small-sized samples, hereby exemplified by the oocytes. Bligh & Dyer still seems to be more suitable for a comprehensive lipid profiling, since this approach allows the removal of the proteins and the detection of all lipid classes profiled in this study, including phosphatidylserines (PS), FFA and acyl-carnitines, which were not well detected otherwise. Although this modified pre-analytical approach based on the Bligh & Dyer method represents a viable option for lipid profiling, it will require further research and validation before it is proposed and widely used in different biological matrices. On the other hand, we acknowledge that for studies on specific subclasses (target lipid extraction), one-step extraction using pure methanol may represent a valid choice and a useful strategy for target lipid extraction tailored to specific biological matrices (e.g., oocytes, embryos, cell lines) and sampling (e.g., microextraction). Finally, the impact of transportation of lipid extracts under vacuum at room temperature was not observed by PCA analysis, meaning that lipid profiling can be performed after room temperature transportation without extensive consequences to the final results.

## Data Availability

Data is available upon request to the corresponding author
